# Supplementary material for: Presurgical Executive Functioning in Low-Grade Glioma Patients Cannot Be Topographically Mapped
Source: Cancers (Basel). 2023 Jan 28;15(3):807. doi: 10.3390/cancers15030807 (PMC9913560; doi:10.3390/cancers15030807)
Supplement: Supplementary file 1 [file cancers-15-00807-s001.zip › cancers-2075221-supplementary.pdf]

Supplementary Tables

Supplementary Table S1. A Spearman’s rank-order correlation for the Tilburg sample

	Minimal distance											
	SLF I				SLF II				SLF III			
	Right (*n*=47)		Left (*n*=50)		Right (*n*=48)		Left (*n*=50)		Right (*n*=47)		Left (*n*=50)	
Cognitive tests:	**rho**	***p* <**	**rho**	***p* <**	**rho**	***p* <**	**rho**	***p* <**	**rho**	***p* <**	**rho**	***p* <**
Shifting attention	-.020	.891	**.410**	**.003***	.047	.752	**.378**	**.007****	.243	.097	.209	.146
Stroop interference	.082	.583	.116	.420	-.092	.537	.162	.260	-.298	.042*	.187	.193
Letter fluency[1]	.061	.703	.726	.055	.246	.122	-.001	.994	.041	.797	-.062	.695
Digit span forward[2]	.028	.895	-.277	.211	-.100	.642	.006	.978	-.079	.715	.175	.437
Digit span backward[3]	.155	.471	-.022	.922	-.001	.997	.105	.642	-.222	.298	.075	.739
	**Mean diffusivity**											
	SLF I				SLF II				SLF III			
	Right (*n*=45)		Left (*n*=49)		Right (*n*=47)		Left (*n*=50)		Right (*n*=47)		Left (*n*=50)	
Cognitive tests:	**rho**	***p* <**	**rho**	***p* <**	**rho**	***p* <**	**rho**	***p* <**	**rho**	***p* <**	**rho**	***p* <**
Shifting attention	-.049	.747	.060	.680	-.067	.649	.089	.537	-.072	.628	.003	.985
Stroop interference	-.014	.925	.317	.146	.162	.275	-.002	.988	.123	.410	.007	.959
Letter fluency[1]	-.141	.393	.007	.963	-.225	.157	-.029	.855	-.157	.328	-.054	.733
Digit span forward[2]	.154	.493	.499	.021	-.024	.910	.193	.391	-.099	.645	.137	.543
Digit span backward[3]	.301	.174	.355	.155	.202	.344	.133	.556	.097	.654	.038	.867

** meaning $p < .01$; * meaning $p < .05$; In bold p < BH-corrected alpha of 0.1;

[1] Data missing right for *n*=6 and left for *n*=7.

[2]Data missing right for *n=23* and left for *n=28*

[3]Data missing right for *n=23* and left for *n=28*

Supplementary Table S1. B Spearman’s rank-order correlation for the Paris sample

	Minimal distance											
	SLF I				SLF II				SLF III			
	Right (*n*=25)		Left (*n*=30)		Right (*n*=25)		Left (*n*=30)		Right (*n*=25)		Left (*n*=30)	
Cognitive tests:	**rho**	***p* <**	**rho**	***p* <**	**rho**	***p* <**	**rho**	***p* <**	**rho**	***p* <**	**rho**	***p* <**
Shifting attention	-.291	.189	.259	.167	-.109	.629	.293	.116	.009	.969	**.439***	**.015**
Stroop interference[1]	-.355	.089	.155	.430	-.307	.145	.130	.510	-.160	.454	-.100	.613
Letter fluency	.183	.403	.154	.418	.363	.089	.178	.347	.424*	.044	.242	.197
Digit span forward	-.238	.252	.184	.329	-.269	.194	.174	.358	.040	.850	.127	.504
Digit span backward	-.142	.500	.163	.388	-.207	.321	.133	.483	-.083	.693	-.123	.517
	**Mean diffusivity**											
	SLF I				SLF II				SLF III			
	Right (*n*=25)		Left (*n*=30)		Right (*n*=25)		Left (*n*=30)		Right (*n*=25)		Left (*n*=30)	
Cognitive tests:	**rho**	***p* <**	**rho**	***p* <**	**rho**	***p* <**	**rho**	***p* <**	**rho**	***p* <**	**rho**	***p* <**
Shifting attention	.335	.128	-.075	.694	.298	.179	-.116	.541	.382	.079	-.117	.539
Stroop interference[1]	.181	.398	.044	.826	.061	.777	-.018	.927	.091	.674	-.038	.848
Letter fluency	-.186	.395	.044	.817	-.366	.086	.038	.842	-.153	.487	.050	.793
Digit span forward	-.173	.408	.097	.612	-.026	.903	.317	.087	-.003	.990	.201	.288
Digit span backward	-.047	.825	.031	.869	.024	.908	.129	.496	.014	.948	-.071	.709

** meaning $p < .01$; * meaning $p < .05$; In bold p < BH-corrected alpha of 0.1;

[1] Data missing right for *n*=1 and left for *n*=2.

Supplementary Table S1. C Spearman's rank-order correlation for the merged sample

	**Minimal distance**											
	**SLF I**				**SLF II**				**SLF III**			
	Right (*n*=71)		Left (*n*=80)		Right (*n*=71)		Left (*n*=80)		Right (*n*=71)		Left (*n*=80)	
Cognitive tests:	**rho**	***p* <**	**rho**	***p* <**	**rho**	***p* <**	**rho**	***p* <**	**rho**	***p* <**	**rho**	***p* <**
Shifting attention[1]	-.082	.499	**.328****	**.003**	-.042	.727	**.365****	**.001**	.191	.113	**.278***	**.012**
Stroop interference[2]	-.047	.699	.125	.276	-.166	.166	.155	.175	-.231	.052	.085	.460
Letter fluency[3]	.104	.412	.102	.392	.254*	.042	.084	.482	.173	.172	.113	.342
Digit span forward[4]	-.116	.427	-.068	.632	-.150	.303	.027	.848	-.016	.915	.120	.396
Digit span backward[5]	-.016	.914	.048	.737	-.056	.704	.071	.615	-.109	.455	-.030	.835
	**Mean diffusivity**											
	**SLF I**				**SLF II**				**SLF III**			
	Right (*n*=69)		Left (*n*=79)		Right (*n*=71)		Left (*n*=80)		Right (*n*=71)		Left (*n*=80)	
Cognitive tests:	**rho**	***p* <**	**rho**	***p* <**	**rho**	***p* <**	**rho**	***p* <**	**rho**	***p* <**	**rho**	***p* <**
Shifting attention[1]	-.029	.817	.058	.612	-.085	.482	.046	.687	-.078	.523	.020	.858
Stroop interference[2]	.130	.289	.199	.083	.164	.173	.089	.439	.150	.212	.119	.301
Letter fluency[3]	-.135	.296	.025	.833	-.248*	.048	.002	.984	-.170	.180	.024	.840
Digit span forward[4]	-.027	.857	.329*	.018	-.034	.819	.326*	.018	-.035	.812	.231	.099
Digit span backward[5]	.089	.550	.165	.247	.099	.550	.159	.260	.030	.837	.002	.991

** meaning $p < .01$; * meaning $p < .05$; In bold p < BH-corrected alpha of 0.1;

[1] Data missing right for *n*=1 and left for *n*=0

[2] Data missing right for *n=0* and left for *n=2*

[3] Data missing right for *n=7* and left for *n=7*

[4] Data missing right for *n=21* and left for *n=18*

[5] Data missing right for *n=21* and left for *n=18*

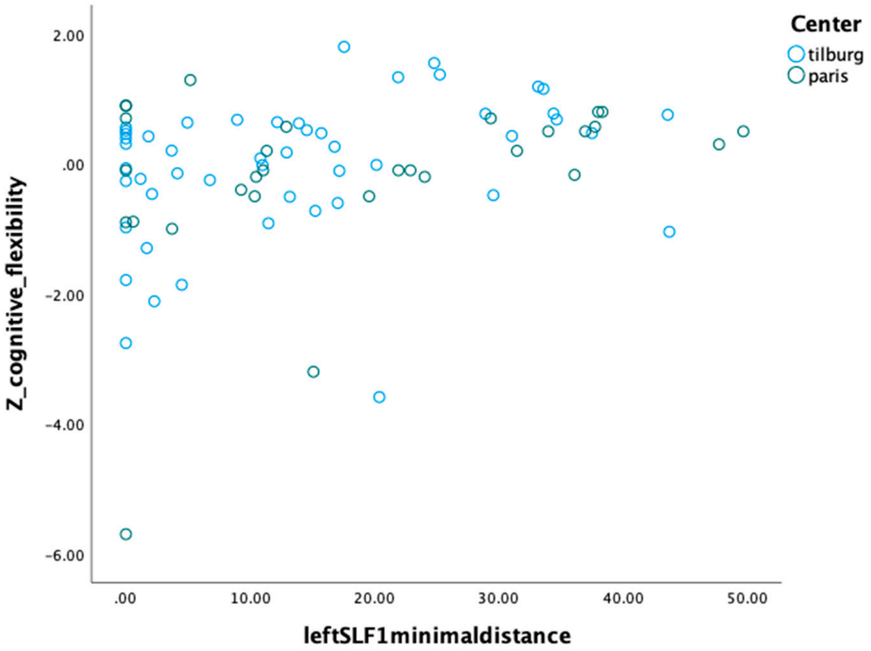

Center
tilburg
paris
Z_cognitive_flexibility
2.00
.00
-2.00
-4.00
-6.00
.00
10.00
20.00
30.00
40.00
50.00
leftSLF1minimaldistance

(**A**)

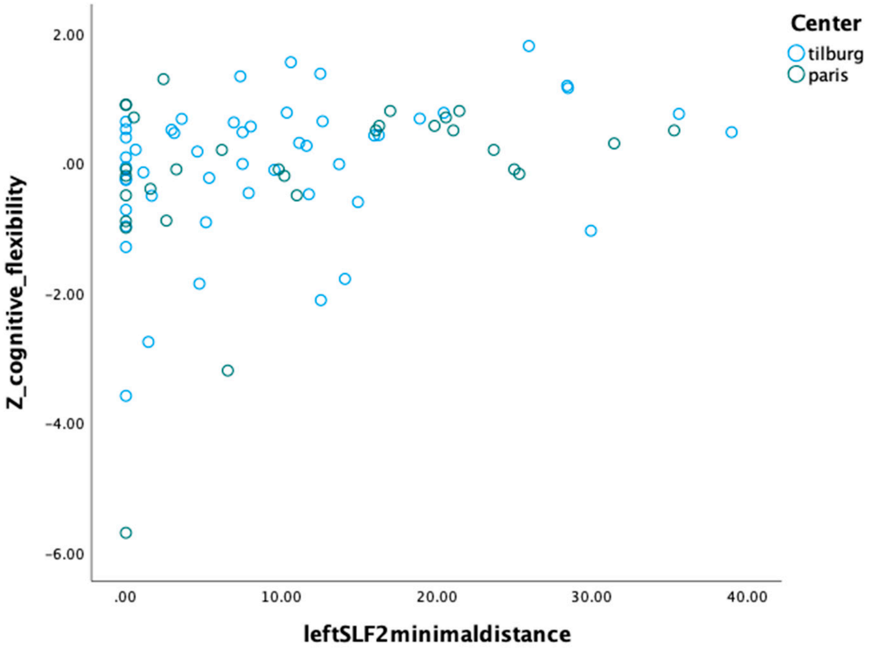

Center
tilburg
paris
Z_cognitive_flexibility
2.00
.00
-2.00
-4.00
-6.00
.00
10.00
20.00
30.00
40.00
leftSLF2minimaldistance

(**B**)

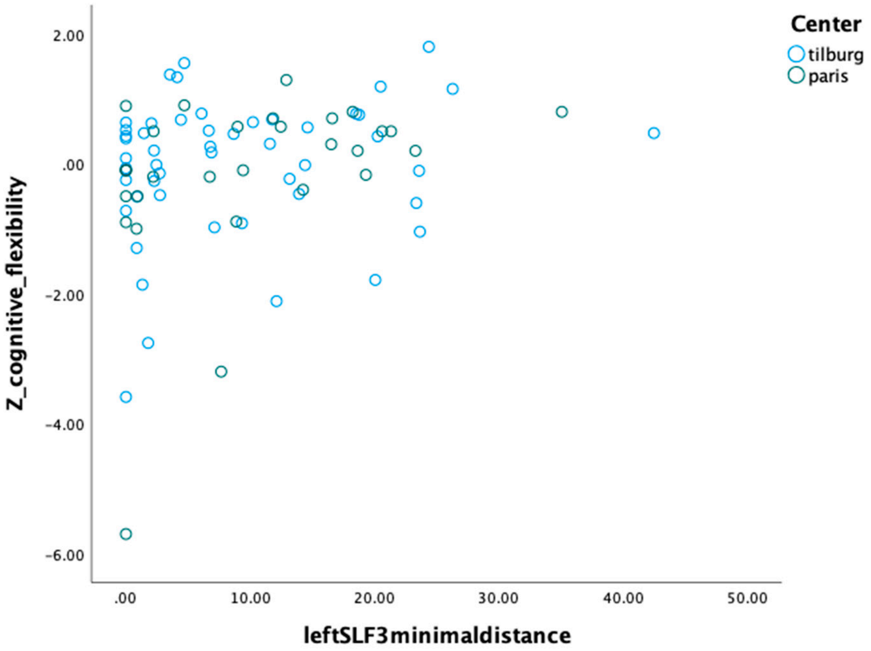

(C)

Figure S1. (A). **Scatterplot for minimal distance of the left SLF I (X-axis) and z-scores of cognitive flexibility** (TMT for Paris sample and Shifting attention task for Tilburg sample). (B). **Scatterplot for minimal distance of the left SLF II (X-axis) and z-scores of cognitive flexibility** (TMT for Paris sample and Shifting attention task for Tilburg sample). (**C**). **Scatterplot for minimal distance of the left SLF III (X-axis) and z-scores of cognitive flexibility** (TMT B-A for Paris sample and Shifting attention task Tilburg sample).
